# Submacular hemorrhage secondary to congenital toxoplasmosis

**DOI:** 10.1590/S1679-45082014RC2708

**Published:** 2014

**Authors:** Ana Luiza Fontes de Azevedo Costa, Thiago Gonçalves dos Santos Martins, Francisco Javier Solano Moncada, Mário Martins dos Santos Motta

**Affiliations:** 1 Hospital Federal dos Servidores do Estado, Rio de Janeiro, RJ, Brazil; 2 Universidade Federal de São Paulo, São Paulo, SP, Brazil; 3 Universidade de São Paulo, São Paulo, SP, Brazil

**Keywords:** Toxoplasmosis, congenital/etiology, Retinal hemorrhage/etiology, Intravitreal injections/adverse effects, Retinal neovascularization/complications, Angiogenesis inhibitors, Tomography, optical coherence, Case reports

## Abstract

We report the case of a patient with congenital toxoplasmosis and submacular hemorrhage caused by a neovascular membrane who underwent an intravitreal injection of C3F8 and bevacizumab, and had a good visual recovery.

## RESUMO

Relatamos o caso de uma paciente com toxoplasmose congênita e hemorragia submacular por uma membrana neovascular submetida à injeção intravítrea de C3F8 e bevacizumabe, com boa recuperação visual.

## INTRODUCTION

The retinochoroiditis is the most common manifestation of congenital toxoplasmosis that is bilateral in 50 to 80% of cases. This inflammation could also compromise the retinal vascular system and the formation of subretinal neovascular membrane, which could be seen in the edge of the lesion or distant from the edge with nourishing arteries arising from the scar.^(1)^

We report a case of patient with congenital toxoplasmosis caused by subretinial neovascular membrane with a submacular hemorrhage that, although infrequent, could promote important consequences because of the visual acuity reduction. Despite this fact, it could be treated with possibility of recovering.

## CASE REPORT

LSE, an 18-year-old student woman was admitted to the *Hospital das Clínicas da Faculdade de Medicina da Universidade de São Paulo* on February 2010, with low visual acuity for 1 week. The patient was diagnosed with ocular congenital toxoplasmosis by the age 15 years old.

**Figure 1:**
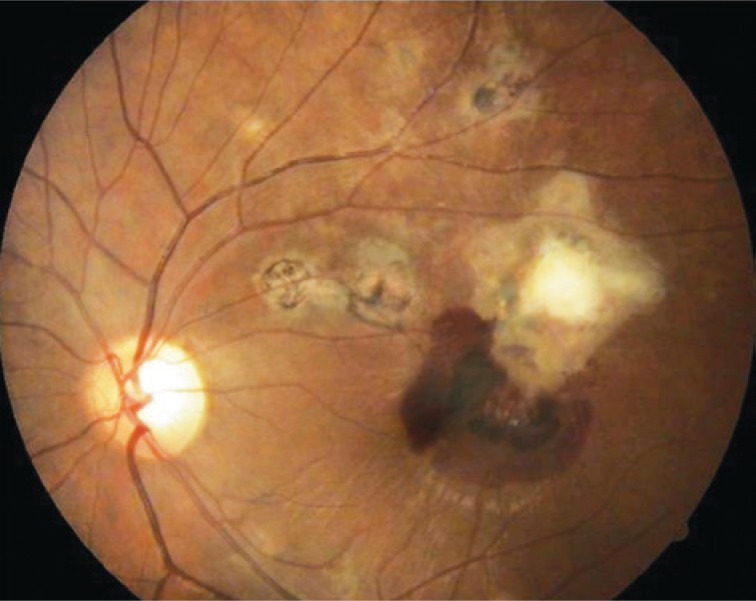
Scar area with submacular hemorrhage in the left eye

An ophthalmologic exam performed at admission showed corrected visual acuity with counting finger from 1m in the right eye and hand motion in the left eye. The visual acuity in the left eye of the patient was 20/20 without correction, which happened before she develops the reported episode according to information collected from the medical record. In order to register and support the diagnosis a retinography and an angiofluoresceinography were conducted (Figure 1). On February 18, 2010 the patient, in face-down position, received an intravitreal injection of 0.3mL of C3F8 in left eye.

**Figure 2:**
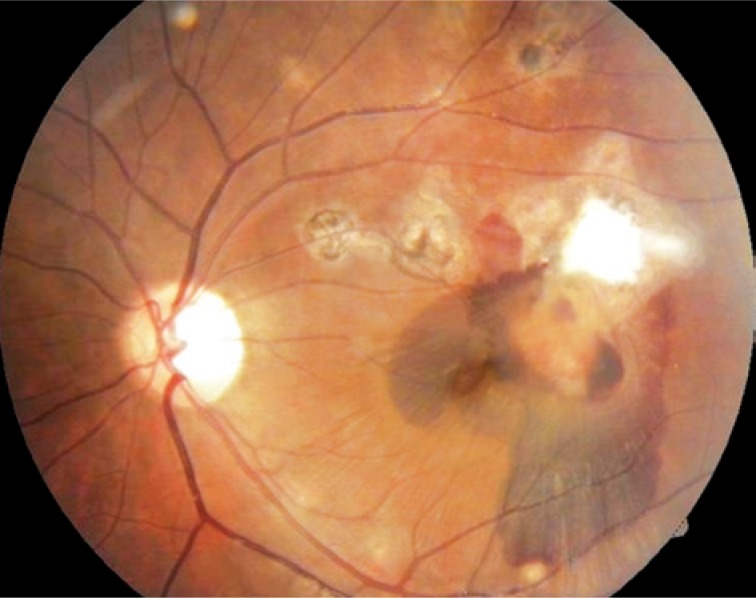
First day after intravitreal injection of C3F8

One day after the procedure the patient was presenting vision of 20/50 and inferior displacement of submacular hemorrhage (Figure 2).

**Figure 3:**
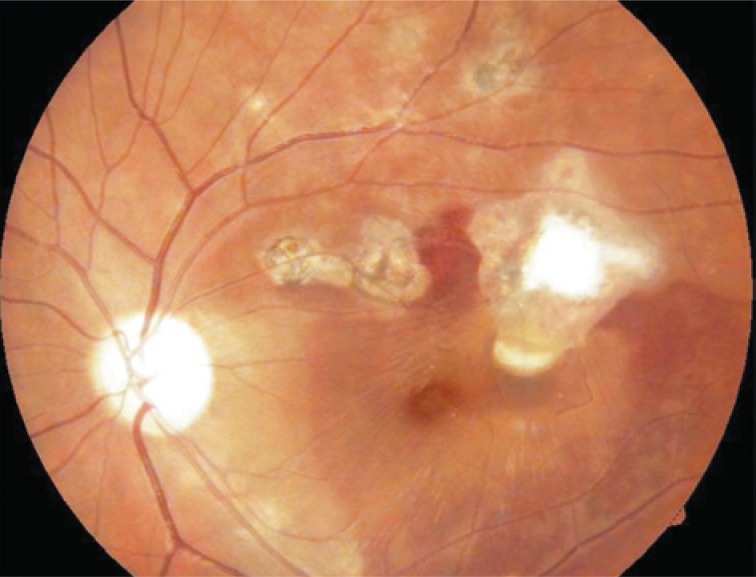
One week after intravitreal injection of C3F8

On August 25, 2010 a reduction of visual acuity to 20/200 was seen. The funduscopy revealed partial absorption of hemorrhage but with macular edema (Figure 3). Based on this clinical feature, on March 1, 2010 an intravitreal bevacizumab injection was conducted.

**Figure 4:**
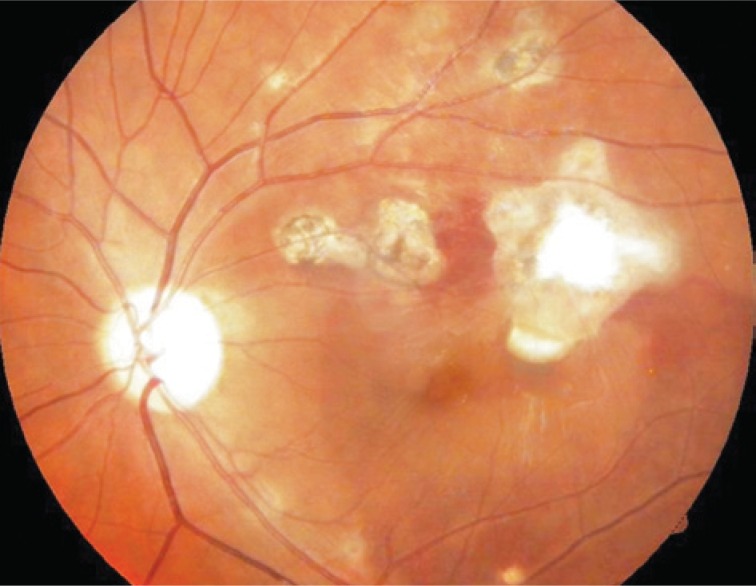
One week after intravitreal injection of bevacizumab

A week after this procedure the patient showed visual acuity of 20/20 in the left eye. The retinography showed a higher reduction of submacular hemorrhage and and hyperfluorescence due to impregnation of neovascular membrane without significant leakage. The optical coherence tomography found a neovascular membrane in front of pigment epithelium without significant intraretinal edema (Figure 4).

**Figure 5:**
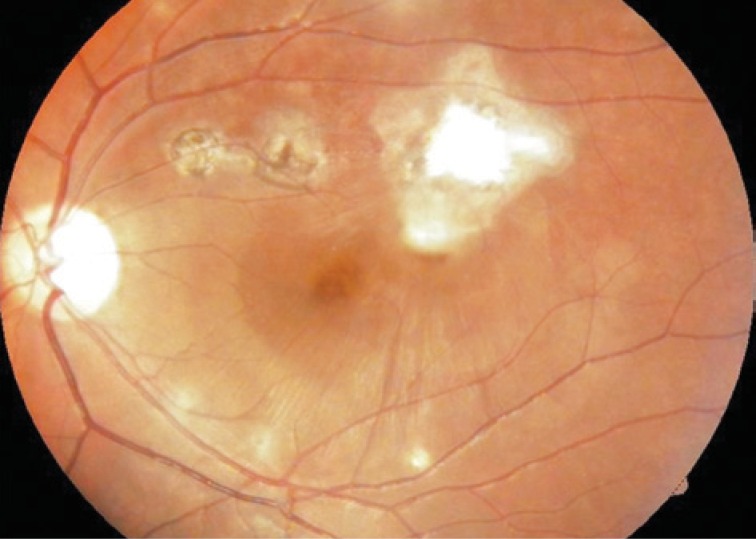
Six months after intravitreal injection of bevacizumab

After 6 months of follow-up, the visual acuity of 20/20 in the left eye was maintained and no signs of membrane activity were found in fundoscopy, fluorescein angiography and optical coherence tomography (Figure 5).

## DISCUSSION

Choroidal neovascular membrane is a rare complication of toxoplasmic chorioretinitis related to the production of vascular endothelial growing factor.^(2)^

Studies that analyzed the causes of submacular hemorrhage found that those secondary to age-related macular degeneration (ARMD) present a worse visual prognosis than those secondary to trauma, macroaneurysm or congenital toxoplasmosis.^(3)^

The treatment with low doses (18 to 50mg) of intravitreal tissue plasminogen activator and 0.3mL perfluoropropane followed by face-down position for 3 days, present good results in patients with submacular hemorrhage secondary to ARMD.^(4)^

There are studies showing that children with neovascular membrane had better visual recovery than adults.^(5)^

A study with intravitreal injection of C3F8 and bevacizumab, the same injection used in our case, reported good visual recovery, probably because it removed the blood in macular region, which decreases the toxicity to photoreceptors.^(6)^ The blood in macular region that come in contact with photoreceptors leads to edema of these receptors after 1 hour of bleeding and causes the destruction of external nuclear layer in 2 weeks due to toxic effects of iron in the hemoglobin.^(7)^ The objective of bevacizumab use is to reduce the vascular permeability that provokes an involution of subretinal neovascular membrane.^(6)^

Other method reported in the literature for treatment of submacular hemorrhage secondary to congenital toxoplasmosis, which presented good results, was the photodynamic therapy.^(8)^

## CONCLUSION

Although unusual, the submacular hemorrhage must be considered in patients with congenital toxoplasmosis who already had scars and subretinal membrane with good visual acuity that evolved with sudden decrease of vision. The lack of studies comparing natural evolution of neovascular membrane secondary to toxoplasmosis scar in the eye enables to state that the best treatment for retinochoroiditis would be the one reported in this case especially for young patients with normal vision in only one eye.
